# Laboratory-Based Surveillance of COVID-19 in South Batinah, Oman, March–July 2020

**DOI:** 10.1007/s44229-022-00008-9

**Published:** 2022-05-31

**Authors:** Zayid K. Almayahi, Nawal Al Kindi, Nasser Al Shaqsi, Noaman Al Hattali, Azza Al Hattali, Khalid Al Dhuhli, Mark E. Beatty

**Affiliations:** 1 Disease Surveillance and Control Department, General Directorate for Health Services, MOH, South Batinah Governorate, P.O. Box 543, 329 Rustaq, Oman; 2 Central Public Health Laboratory, Directorate General for Disease Surveillance and Control, MOH, Muscat, Oman; 3 Epidemiology and Public Health Consulting, LLC, Everett, WA USA

**Keywords:** Oman, COVID-19, Surveillance, Testing, SARS CoV-2, Laboratory, First wave, Pandemic, Case definition

## Abstract

**Objective:**

The successful response to COVID-19 would require an effective public health surveillance and management, technical expertise, and smart mobilization of many resources. This study aimed to analyze COVID-19 epidemiological profile with respect to the changing case definitions and testing performance.

**Methods:**

Data were extracted from the electronic notification system (Tarassud) from 1 January to 13 July 2020. The information used was primarily composed of details regarding samples, age, sex, nationality, residence and hospital admission. Bivariate and multivariable analyses were used to determine the odds ratios (ORs) and 95% confidence intervals (CI).

**Results:**

A total of 20,377 COVID-19 tests were performed from 15 March to 13 July 2020. Most (4885; 87.2%) positive tests were among Omanis, and 3602 (64.3%) were in males. The median age of people tested was 30 (standard deviation 16.5) years (interquartile range 22, 38). The odds of acquiring infection increased with age. The ORs of infection for groups of 30–39, 40–49 and over 50 years of age were 2.75 (95% CI 2.42–3.13), 3.29 (95% CI 2.85–3.79) and 3.34 (95% CI 2.89–3.87), respectively. Likewise, admission rates increased with age; the ORs for the groups 40–49 and ≥ 50 years of age were 4.45 (95% CI1.35–14.67) and 16.53 (95% CI 5.18–52.75), respectively. Multivariate analysis identified Barka 1.4 (95% CI 1.33–2.27) and Al Musanaah 1.4 (95% CI 1.07–1.84) as having the highest risk of transmission. Of 5604 people with positive results, 160 (2.9%) required hospital admission, and males had higher odds of admission, with an OR of 1.5 (95% CI 1.05–2.13). The average delay in the release of test results further increased after the fourth and fifth case definitions were adopted (2.04 and 2.56 days, respectively).

**Conclusion:**

Age was a significant factor associated with infection and hospital admission. Transmission occurred mainly among Omanis, and Barka and Al Musanaah reported the highest rates of transmission. Prioritization of testing accessibility should continually be assessed for high-risk groups, particularly when resources become limited.

## Background

Although it has been a year at the time of writing this report since the early signals of a mysterious and unknown viral infection causing pneumonias and deaths in China, the challenges posed by the coronavirus disease 2019 (COVID-19) pandemic are intensifying. The pandemic has had profound effects on many aspects of life. Severe acute respiratory syndrome coronavirus 2 (SARS-CoV-2), which first emerged in Wuhan, China, quickly showed unpredictable and non-containable global spread and caused a startling number of fatalities [1]. As of 30 December 2020, the virus had infected more than 80 million people globally, causing 1,771,128 deaths, whereas Oman had recorded 128,563 cases and 1495 deaths [2].

Countries’ responses have markedly varied, reflecting a range of risk caused by the pandemic. Moreover, the exact strategy adopted by each country has been greatly influenced by economic resources and existing health and medical services [3]. Subsequently, the burden on health systems, and the number of cases of infection and deaths have varied among countries. For instance, countries such as New Zealand, which considered COVID-19 a serious threat and took early and strict action, fared better than many other countries [4].

Moreover, many countries had months to prepare after the emergence of SARS-CoV-2 in China, had reasonably good economic funding and implemented preventive measures as early as possible, yet experienced rapid community transmission and exponential surges in cases. Thus, successful containment appears to rely on several major factors including the timing and types of adopted preventive measures, sociodemographic characteristics, testing, availability and accessibility of medical services, national economic resources, and the resilience and management characteristics of decision-makers and leading authorities [5–8].

Analysis of laboratory-based surveillance in South Batinah Governorate (SBG), the fifth most populous Omani governorate, reflects a situation similar to that of the entire country. The Ministry of Health (MOH) used several case definitions, which changed according to the evolving epidemiology of the disease. Despite the changing case definitions and the high level of testing, extensive contact tracing and other preventive measures, the number of cases rose rapidly.

We analyzed the surveillance data to describe the epidemiology of cases and hospital admissions in SBG, considering the case definitions used and the testing performance.

## Methods

SBG is located on the shores of the Gulf of Oman, west of Muscat, east of North Batinah and north of the Dakhilyah governorates. It includes six states (called wilayats): Barka, Musanaah, Rustaq, Nakhal, Wadi Al Mawel, and Awabi. Governmental medical services are provided by 21 primary health centers, two preventive medical centers, and one secondary hospital. The provincial department of disease surveillance and control is responsible for managing communicable diseases in the governorate, including the COVID-19 disease. The MOH’s case definition for suspected case of COVID-19 was modified five times since the beginning of the pandemic, according to the epidemic status in the country (Table 1).

**Table 1:** Timeframe for the five definitions of suspected cases and the corresponding number of tests and positive cases

Case definition number	Definition	Date of release	No. of days	No. of tests	No. of positive cases
1	A suspected case is that in a person with severe acute respiratory infection (SARI), with a history of fever and cough requiring admission to the hospital with one of the following: (1) travel to China in the past 14 days, (2) development of an unusual or unexpected clinical course, particularly sudden deterioration despite appropriate treatment, (3) close physical contact with a person with a confirmed case of 2019-nCoV infection, while that person was symptomatic and (4) visiting a healthcare facility in a country where hospital associated 2019-nCoV infections have been reported	30 January 2020	25	0	0
2	A suspected case is that in (A) a person with acute respiratory infection (sudden onset of at least one of the following: fever, cough or shortness of breath) AND who, in the 14 days prior to the onset of symptoms, met at least one of the following epidemiological criteria: (1) travel to affected countries (check Ministry of Health website for updated list of countries or call the Governorate hotline) OR (2) close contact with a person with a confirmed case of COVID-19 infection; (B) a patient admitted with severe acute respiratory infection (SARI) AND with no other etiology that fully explains the clinical presentation	24 February 2020	21	1	0
3	A suspected case is that in (A) a person with acute respiratory infection (sudden onset of at least one of the following: fever ≥ 38 °C, cough, shortness of breath or sore throat) AND in the 14 days prior to onset of symptoms, met at least one of the following epidemiological criteria: (1) a history of international travel OR (2) a history of contact with a cluster (two or more cases with fever and acute respiratory symptoms in a small area, e.g., within families, offices or schools) OR (3) close contact with a person with a confirmed case of COVID-19 OR (4) health care work involving direct medical management of patients with respiratory symptoms in a hospital setting; (B) a patient admitted with community acquired pneumonia; (C) a patient admitted with severe acute respiratory infection (SARI) or one who develops SARI in the hospital	16 March 2020	21	353	18
4	A suspected case is that in (A) a person with acute respiratory symptoms AND/OR (B) a person with pneumonia AND/OR (C) a patient admitted with severe acute respiratory infection (SARI) or one who develops SARI in the hospital	6 April 2020	61	9724	1045
5	A suspected case is almost same as that in case definition number 4, with an added definition of probable cases (related to clustering) in which testing is not performed. Clustering defined as follows: Workplace/dormitories: if three coworkers/inmates staying in one place are lab-confirmed positive (inform the Department of Disease Surveillance and Control of the Governorate). Families: if three people are lab-confirmed positive in one family staying together in one house. The same is applied for shared accommodations *High risk symptomatic individuals are tested even if they are classified as having probable cases	16 June 2020	37	10,528	4541

Initially, manual notifications were sent from the reporting health facilities by fax, email or mobile phone text message. By the end of April 2020, the department started to receive electronic notifications through the Tarassud system, the national electronic surveillance system of the MOH, which was launched on 3 May 2017 [9]. It is a flexible web-based system used for receiving notifications regarding communicable diseases from all health institutions in the MOH. However, during the COVID-19 pandemic, the Tarassud system was upgraded and extended to support notifications of suspected and confirmed cases of COVID-19 from all medical governmental and private institutions through protected and secured online web-based access. Beyond providing notifications, the system is used for other purposes, including intelligent tracking of quarantined people, registration of quarantined people from different ports, and provision of immunization and laboratory survey results. The COVID-19 test results reported before the implementation of Tarassud were entered into the Tarassud system.

The entered data included demographic data, exposure and contact information, clinical presentation, and laboratory results. The data were then exported from the Tarassud system into Excel (Microsoft, Redmond, WA) for analysis.

The analysis was limited to reports in Tarassud from 1 January 2020 to 13 July 2020 from South Batinah healthcare facilities. A reporting delay created an artifact of fewer totals from 8 to 13 July.

At first, all samples from the governorate were processed in the Central Public Health Laboratory (CPHL) in Muscat. The CPHL performed testing through reverse transcriptase polymerase chain reaction (RT PCR). Several kits were used depending on availability, but all were internationally approved for SARS-CoV-2 testing (liferiver, Changsha, China, Fast Track Diagnostics, Sansure and Kingfisher kits). A fully automated (real-time) Cobas6800 (Roche) instrument was used for most testing. In late April, the secondary hospital began point-of-care testing with Xpress SARS-CoV-2/GeneXpert (Cepheid), a qualitative test that detects specific viral nucleic acids and proteins [10]. The assay had received emergency use authorization from the US Food and Drug Administration (FDA) on 23 March 2020 [10, 11]. However, testing in the hospital was limited to people with suspected cases requiring admission and to health care workers. The results were intended to be reported in less than 24 h; however, as samples accumulated during the community transmission stage, results were reported late. The criteria for admission were a positive PCR test, and a clinical presentation of pneumonia with substantial blood oxygen desaturation.

A total of 20,689 results were included in the system. However, 52 tests were repeats on the same patient on different days; 28 tests were performed for the same episode of symptoms in different facilities; and 3 results on the same patient were classified as inconclusive (only one gene positive without clinical indications of COVID-19). In the case of multiple results for the same patient, only the first positive result was included in the analysis. Thus, the total number of results decreased to 20,606. Of these, 34 samples were inconclusive, 163 were rejected, and 32 samples were not run. Therefore, 20,337 results on individual patients remained for analysis: 5604 were positive, and 14,773 were negative.

Two software packages, Excel (Microsoft, Redmond, WA) and SPSS 23.0 (IBM Corp., Armonk, NY), were used to organize, tabulate, and analyze the data. We calculated the odds ratios (OR) and 95% confidence intervals (CI) with bivariate and multivariate analyses. A *P*-value ≤ 0.05 was considered statistically significant.

## Results

A total of 20,377 test results from 15 March to 13 July 2020 were collected. The majority (4885; 87.2%) of positive tests were among Omanis, and 3602 (64.3%) were in males. Occupation data were available for only 7310 (35.5%) patients. Most (1037; 14.2%) had office, managerial, teaching, or computer jobs, almost 17% were unemployed, and 5.1% were health care workers.

The median age of those tested was 30 (standard deviation [SD] 16.5) years (interquartile range [IQR] 22, 38), whereas the median age for patients with positive tests was 32 (SD 14.8) years (IQR 25, 41). Patients 20–39 years of age accounted for 58.9% of the total positive tests, whereas those younger than 10 years of age had the lowest positivity rate (6.1%). With the age category < 10 years as a reference group, the odds of acquiring the infection increased with the age. The ORs for groups 20–29, 30–39, 40–49 and over 50 years of age were 2.62 (95% CI 2.4–3.07), 2.49 (95% CI 2.19–2.83), 3.29 (95% CI 2.85–3.79) and 3.34 (95% CI 2.89–3.87), respectively. With 316 test results performed on patients living outside SBG used as a reference group, the odds of a positive test result were highest for patients living in Barka, at 1.4 (95% CI 1.33–2.27), and Al Musanaah, at 1.4 (95% CI 1.07–1.84). The findings also revealed that non-Omanis had a lower likelihood of infection than Omanis, at OR = 0.884 (0.802–0.973; Table 2).

**Table 2:** Age, sex and nationality of people tested for SARS-CoV-2 in South Batinah from 15 March to 13 July 2020

Variable	Positive *N* = 5604	Negative *N* = 14,473	OR (95% CI) Bivariate analysis	OR (95% CI) Multivariable analysis
	No. (%)	No. (%)		
Age (years)				
< 10 (reference group)	344 (6.1)	2396 (16.2)	1	1
10–19	449 (8)	1139 (7.7)	2.75 (2.35–3.21)	2.62 (2.4–3.07)
20–29	1577 (28.1)	4137 (28)	2.66 (2.34–3.02)	2.49 (2.19–2.83)
30–39	1726 (30.8)	4067 (27.5)	2.96 (2.61–3.35)	2.75 (2.42–3.13)
40–49	819 (14.6)	1640 (11.1)	3.48 (3.02–4.00)	3.29 (2.85–3.79)
≥ 50	689 (12.3)	1394 (9.4)	3.44 (2.98–3.98)	3.34 (2.89–3.87)
Median + SD (IQR)	32 + 14.8 (25, 41)	29 + 17.0 (20, 37)		
Sex				
Female	2002 (35.7)	5435 (36.8)	1	1
Male	3602 (64.3)	9338 (63.2)	1.05 (0.98–1.12)	1.03 (0.96–1.1)
Nationality				
Omani	4885 (87.2)	12,941 (87.6)	1	1
Non-Omani	719 (12.8)	1832 (12.4)	1.04 (0.945–1.14)	0.884 (0.802–0.973)
Wilayat				
Barka	2544 (45.4)	4927 (33.4)	1.7 (1.3–2.2)	1.4 (1.33–2.27)
Al Musanaa	1171 (20.9)	2734 (18.5)	1.4 (1.1–1.8)	1.4 (1.07–1.84)
Ar Rustaq	1140 (20.3)	4440 (30.1)	0.84 (0.6–1.1)	0.9 (0.68–1.17)
Awabi	198 (3.5)	848 (5.7)	0.76 (0.56–1.03)	0.83 (0.61–1.13)
Nakhal	229 (4.1)	881 (6)	0.85 (0.63–1.15)	0.923 (0.68–1.25)
Wadi Mawel	248 (4.4)	701 (4.7)	1.16 (0.86–1.56)	1.29 (0.95–1.74)
Outside South Batinah (reference group)	74 (1.3)	242 (1.6)	1	1

*CI* confidence interval, *IQR* interquartile range, *OR* odds ratio, *SARS-CoV-2* severe acute respiratory syndrome coronavirus 2, *SD* standard deviation

Of 5604 people with positive results, 160 (2.9%) required hospital admission (no readmission or duplication). Approximately two-thirds (107; 66.9%) were males, and 140 (87.5%) were Omanis. More than half (84; 52.5%) of the cases were in people 50 years of age or older. By contrast, only five (3.2%) of those admitted were under 20 years of age. With the < 10 year age category used as a reference group, the admission rates increased with age; the Ors for the age groups 40–49 and ≥ 50 were 4.45 (95% CI1.35–14.67) and 16.53 (95% CI 5.18–52.75), respectively. Males had a greater likelihood of admission than females, with an OR of 1.5 (95% CI 1.05–2.13). Almost half (79; 49.4%) of the admitted patients had their results available on the same day, and only three (1.9%) had their results released in 5 days or more (Table 3).

**Table 3:** Age, sex, nationality, and time for release of results for admitted vs. non-admitted patients in South Batinah until 13 July 2020

Variable	Admitted (*N* = 160)	Not admitted (*N* = 5444)	OR (95% CI) Bivariate analysis	OR (95% CI) Multivariable analysis
	No. (%)	No. (%)		
Age (years)				
< 10 (reference group)	3 (1.9)	341 (6.3)	1	1
10–19	2 (1.3)	447 (8.2)	0.51 (0.09–3.06)	0.51 (0.09–3.08)
20–29	10 (6.3)	1567 (28.8)	0.73 (0.20–2.65)	0.71 (0.2–2.6)
30–39	30 (18.8)	1696 (31.2)	2.01 (0.61–6.63)	1.98 (0.6–6.54)
40–49	31 (19.4)	788 (14.5)	4.47 (1.36–14.73)	4.45 (1.35–14.67)
≥ 50	84 (52.5)	605 (11.1)	15.78 (4.95–50.31)	16.53 (5.18–52.75)
Mean + SD IQR	51.3 + 18.6 (38,67)	32.3 + 14.3 (24,40)		
Sex				
Female	53 (33.1)	1949 (5.8)	1	1
Male	107 (66.9)	3495 (64.2)	1.13 (0.81–1.57)	1.5 (1.05–2.13)
Nationality				
Omani	140 (87.5)	4745 (87.2)	1	1
Non-Omani	20 (12.5)	699 (12.8)	0.97(0.6–1.56)	0.79 (0.48–1.3)
Days until test results (latest test chosen)				
Same day	79 (49.4)	61 (1.1)	127.35 (38.92–416.68)	–
After 1 day	18 (11.3)	436 (8.0)	4.06 (1.19–13.91)	
After 2 days	21 (13.1)	1468 (27.0)	1.41 (0.42–4.75)	
After 3 days	27 (16.9)	2059 (37.8)	1.29 (0.39–4.28)	
After 4 days	12 (7.5)	1125 (20.7)	1.05 (0.29–3.74)	
After 5 days (reference group)	3 (1.9)	295 (5.4)	1	

*CI* confidence interval, *IQR* interquartile range, *OR* odds ratio, *SD* standard deviation

Because the onset dates of disease for most patients were not available, the epidemiologic curve was based on the day of sample collection. Before the fourth case definition went into use on 6 April 2020, the daily average number of samples was 17. After that date, the rate dramatically increased, reaching 386 on 14 June 2020. The first confirmed case of COVID-19 occurred on 20 March 2020. As of 23 May 2020, the daily positivity rate was less than 17%. Two days later, the positivity rate increased to 25%; in less than a month, it remained above 50%. The first admission occurred on 12 April 2020. Daily admissions began to occur on 24 May 2020. The maximum number of admissions in a single day was nine, on 5 July 2020.

**Fig. 1:**
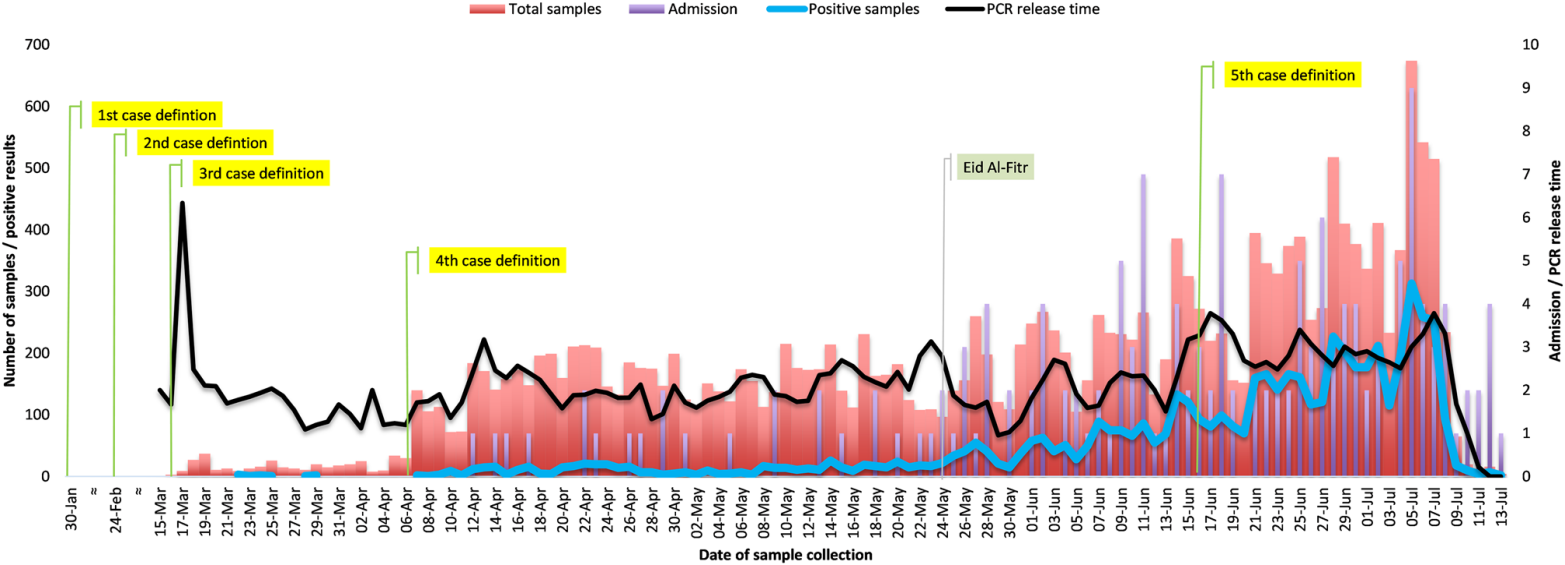
Daily numbers of COVID-19 samples, positivity rate (left axis), daily admission cases, and PCR result release time (right axis)

During the use of the third case definition, the average time between sample collection and result reporting was 1.89 days, with a maximum delay recorded on 17 March 2020, when the CPHL experienced temporary challenges. The average delay further increased after the fourth and fifth case definitions were adopted (2.04 and 2.56 days, respectively). The delay was observed because of an increase in the daily test samples whereas the capability of transporting and processing samples remained unchanged (Fig. 1).

## Discussion

This retrospective analysis of data provided by the electronic health notification system (Tarassud) revealed several important findings. Although people 20–40 years of age accounted for the largest proportion of cases of virus transmission, older people were more prone to developing infection, thus requiring medical care and admission. Non-Omanis showed a 12% decrease in the risk of acquiring the infection compared to Omanis, during the first wave of the COVID-19 pandemic, whereas the risk of severe disease was 50% greater among males than females. Barka and Musanaa wilayats reported the highest rates of transmission in the SBG during the study period.

The preventive measures of institutional quarantine and isolation measures for positive cases, contact tracing, PCR testing and social distancing that were taken at the beginning of the COVID-19 pandemic appeared to substantially contribute to mitigating transmission within the country. However, a delay in reporting of results probably began because the health and public health systems became overwhelmed, particularly after 24 May 2020, when the positivity rate began to increase. Initially the case definition was not sufficiently sensitive and required adaptation according to local epidemiology, thus resulting in an increase in detection. This finding might also have resulted from delayed preventive measures, especially when some early clusters were detected from the beginning [12].

The national seroprevalence survey in Oman indicated that SBG was the second most affected governorate after the capital Muscat in the first survey cycle performed on July 2020, whereas Musanaa was the wilayat with the greatest prevalence at the end of the fourth cycle [13]. The COVID-19 reporting rate per 100,000 population in SBG was 59.4 as of the end of April 2020 [14].

The first 18 positive cases in SBG did not occur until the third version of the case definition was adopted. Whether this finding was due to increased sensitivity or increased transmission cannot be determined. However, these cases were primarily associated with travel, thus suggesting the beginning of transmission in the region. After 3 weeks, the case definition was further modified to include all acute respiratory cases and consequently to ensure that transmission had been truly established in the community. However, the vast heterogeneity in the presentation and sequelae of COVID-19 and related uncertainties forced the public health system to adopt a more sensitive case definition. Nonetheless, a substantial number of infections were probably missed, even with the most sensitive case definition, because of variations in clinical presentation or the occurrence of asymptomatic infections [15].

The findings of this analysis also confirmed that age is a significant factor associated with symptomatic infection and disease severity (on the basis of hospital admission as a proxy for severe disease) particularly among male patients [14, 16–19]. Although the information on occupation was incomplete, the data indicated the wide spread of infection among different sectors in the community, including many unemployed individuals and health care workers [20].

The daily average number of positive cases remained low, with an average of 10, until 24 May 2020, which corresponded to Muslims’ celebration of Eid Al Fitr. The detailed reasons for the rapid surge in cases thereafter include multiple possible explanations, but gatherings due to the holiday might have been the root cause. In addition, the growing number of samples per day probably led to further delays in result reporting and subsequently delayed self-quarantine measures among positive individuals.

Field experience and continual communication with patients indicated suboptimal level of patient compliance with self-quarantine as people with suspected cases waited for their final test results. Many started isolating only after being confirmed as positive. Therefore, reporting delays may have led to additional transmission in the community that might have been preventable. Mathematical modeling of transmission has shown that minimizing testing and tracing delays could prevent as much as 80% of COVID-19 transmission [8]. The increased delay in reporting reflected the increased number of samples requiring testing, which exceeded the capacity to process samples. A strategy of widespread or even universal testing is more effective in diseases in which all infected people are symptomatic, isolation is ensured, and the sensitivity of the tests is reasonably high. Suspected cases must isolate on the day of symptom onset or at least the same day on which blood is sampled for testing, and all contacts must be promptly listed and quarantined [21].

Likewise, the increasing number of samples tested paralleled an increase in admissions to the secondary hospital and therefore is a proxy for community transmission. Almost half the admissions occurred on the same day as sample collection, thus suggesting most patients experienced rapid and severe deterioration. In some cases, samples were collected, but the results were not available while the patient clinically worsened at home. When the patients returned to the hospital, a second sample was collected for priority testing to confirm the diagnosis. Indeed, the duration from the onset of symptoms to hospital admission was previously estimated to be 2–7 days in symptomatic patients [22].

Public health decision-makers should consider prioritizing sample collection in a staged approach that depends on the transmission level in a given community, as has been adopted in other countries to better manage the testing burden. This strategy would help overcome the challenge of limited resources while simultaneously maintaining the ability to control disease spread and provide medical care at the proper time. SBG, for instance, had the highest transmission of cases in Barka and Musanaa. Indeed, the two wilayats (administrative divisions) are geographically close to the Muscat and North Batinah governorates, both of which were the governorates reporting the most COVID-19 cases in Oman at the time of writing of this report [23]. However, the transmission for the rest of the SBG wilayats was more sporadic, and cluster aggregation of cases suggested lower levels of transmission.

The interim guidance published by the World Health Organization on 21 March 2020 (Laboratory testing strategy recommendations for COVID-19) has recommended the absolute need for prioritization of testing, on the basis of the epidemiological situation of each country according to the evidence of community transmission [24].

We believe that regulated or stratified testing based on epidemiological needs, could contribute to better control during the community transmission stage, given that the delay in self-quarantine allowed for additional transmission that in turn overwhelmed testing and health care resources.

Refining the case definitions to prioritize testing for the groups at highest risk of infection or continuing use of the most sensitive case definition only in selective areas (wilayats) where community transmission had not yet been demonstrated might or might not have been options. Whether such measures would have allowed the health care system to test and return results within 2 days is unknown. Patients at risk of developing severe disease and vulnerable people requiring hospitalization, as well as health care workers, require the earliest testing [24]. However, not only testing but also other preventive measures should be focused on to prevent infection among people at high risk [25].

We also expect that adding or upgrading preventive measures, such as testing strategies, case definitions, lockdown and vaccination, would require health systems to be more resilient and flexible. Such substantial modifications of strategies may not be easy to implement. In addition, all medical professionals in both preventive and clinical sectors were facing this novel virus and disease for the first time, with little valid information [26]. Therefore, the various reasonable and valid approaches used in many countries including Oman must be regarded as best attempts based on limited scientific information. Through additional research and learning to better understand the nature of the virus, the best transmission prevention modalities will continue to be refined.

This analysis was limited by the incomplete data in the Tarassud system, particularly regarding sociodemographic aspects. It also did not consider other causes of admission, and it assumed that the main cause was COVID-19, owing to an absence of clinical data for patients. However, respiratory symptoms were the main clinical manifestations, and the results of this analysis are in agreement with the literature.

In conclusion, COVID-19 transmission occurred mainly among Omanis and in certain areas in SBG. Infection and hospital admission were observed with increasing age. Testing strategy remains a major aspect among other important preventive measures to control disease transmission within communities. Prioritization of preventive measures and testing accessibility should continually be assessed and addressed for high-risk groups, particularly when resources become limited.

## Data Availability

Data are available on reasonable request.

## Abbreviations

COVID-19: Coronavirus disease 2019
SARS-CoV-2: Severe acute respiratory syndrome coronavirus 2
SBG: South Batinah Governorate
CPHL: Central Public Health Laboratory
RT PCR: Reverse transcriptase polymerase chain reaction
SD: Standard deviation
OR: Odds ratio

## References

[1] Ke R, Sanche S, Romero-Severson E, Hengartner N (2020). Fast spread of COVID-19 in Europe and the US suggests the necessity of early, strong and comprehensive interventions. medRxiv.

[2] WHO Coronavirus Disease (COVID-19) Dashboard [Internet]. [cited 2020 Dec 30]. Available from: https://covid19.who.int

[3] Pearce N, Lawlor DA, Brickley EB (2020). Comparisons between countries are essential for the control of COVID-19. Int J Epidemiol.

[4] Baker M, Kvalsvig A, Verrall AJ, Telfar-Barnard L, Wilson N (2020). New Zealand's elimination strategy for the COVID-19 pandemic and what is required to make it work. N Z Med J.

[5] Prem K, Liu Y, Russell TW, Kucharski AJ, Eggo RM, Davies N (2020). The effect of control strategies to reduce social mixing on outcomes of the COVID-19 epidemic in Wuhan, China: a modelling study. Lancet Public Health.

[6] Binagwaho A (2020). We need compassionate leadership management based on evidence to defeat COVID-19. Int J Health Policy Manag.

[7] Legido-Quigley H, Asgari N, Teo YY, Leung GM, Oshitani H, Fukuda K (2020). Are high-performing health systems resilient against the COVID-19 epidemic?. Lancet.

[8] Kretzschmar ME, Rozhnova G, Bootsma MCJ, van Boven M, van de Wijgert JHHM, Bonten MJM (2020). Impact of delays on effectiveness of contact tracing strategies for COVID-19: a modelling study. Lancet Public Health.

[9] Public Health Bulletin, Ministry of Health, Oman, volume 1, issue 2, 2017. [Internet]. [cited 2020 Dec 31]. Available from: https://www.moh.gov.om/documents/236878/0/Public+Health+Bulletin+%232/0b20635e-e197-4897-ac00-24644ce6b3e7

[10] Loeffelholz MJ, Alland D, Butler-Wu SM, Pandey U, Perno CF, Nava A (2020). Multicenter evaluation of the Cepheid Xpert Xpress SARS-CoV-2 test. J Clin Microbiol.

[11] Koonin LM (2020). Novel coronavirus disease (COVID-19) outbreak: now is the time to refresh pandemic plans. J Bus Contin Emerg Plan.

[12] COVID-19: Oman puts Muttrah province in lockdown [Internet]. [cited 2020 Dec 31]. Available from: https://gulfnews.com/world/gulf/oman/covid-19-oman-puts-muttrah-province-in-lockdown-1.70747248

[13] Al-Abri SS, Al-Wahaibi A, Al-Kindi H, Kurup PJ, Al-Maqbali A, Al-Mayahi Z (2021). Seroprevalence of SARS-CoV-2 antibodies in the general population of Oman: results from four successive nationwide sero-epidemiological surveys. Int J Infect Dis.

[14] Al-Rawahi B, Prakash KP, Al-Wahaibi A, Al-Jardani A, Al-Harthy K, Kurup PJ (2021). Epidemiological characteristics of pandemic coronavirus disease (COVID-19) in Oman. Sultan Qaboos Univ Med J.

[15] Gao Z, Xu Y, Sun C, Wang X, Guo Y, Qiu S (2021). A systematic review of asymptomatic infections with COVID-19. J Microbiol Immunol Infect.

[16] Al Wahaibi A, Al Rawahi B, Patel PK, Al Khalili S, Al Maani A, Al-Abri S (2021). COVID-19 disease severity and mortality determinants: a large population-based analysis in Oman. Travel Med Infect Dis.

[17] Khamis F, Al-Zakwani I, Al Naamani H, Al Lawati S, Pandak N, Omar MB (2020). Clinical characteristics and outcomes of the first 63 adult patients hospitalized with COVID-19: an experience from Oman. J Infect Public Health.

[18] Viveiros A, Gheblawi M, Aujla PK, Sosnowski DK, Seubert JM, Kassiri Z (2022). Sex- and age-specific regulation of ACE2: insights into severe COVID-19 susceptibility. J Mol Cell Cardiol.

[19] Kang S-J, Jung SI (2020). Age-related morbidity and mortality among patients with COVID-19. Infect Chemother.

[20] Al Mayahi ZK, Al Kindi N, Al Shaqsi N, Al Hattali N, Al Hattali A, Salim K (2021). Non-respiratory droplet transmission of COVID-19 in the isolation ward of a secondary hospital in Oman: a return to isolation basics. Infect Dis Clin Pract.

[21] Cleevely M, Susskind D, Vines D, Vines L, Wills S (2020). A workable strategy for COVID-19 testing: stratified periodic testing rather than universal random testing. Oxf Rev Econ Policy.

[22] Chen J, Qi T, Liu L, Ling Y, Qian Z, Li T (2020). Clinical progression of patients with COVID-19 in Shanghai, China. J Infect.

[23] COVID-19 pandemic in Oman. In: Wikipedia [Internet]. 2020 [cited 2021 Jan 1]. Available from: https://en.wikipedia.org/w/index.php?title=COVID-19_pandemic_in_Oman&oldid=993339748

[24] World Health Organization. Laboratory testing strategy recommendations for COVID-19: interim guidance, 21 March 2020. 2020 [cited 2020 Aug 15]; Available from: https://apps.who.int/iris/handle/10665/331509

[25] Iser BPM, Sliva I, Raymundo VT, Poleto MB, Schuelter-Trevisol F, Bobinski F (2020). Suspected COVID-19 case definition: a narrative review of the most frequent signs and symptoms among confirmed cases. Epidemiol Serv Saude.

[26] Umakanthan S, Sahu P, Ranade AV (2020). Origin, transmission, diagnosis and management of coronavirus disease 2019 (COVID-19). Postgrad Med J.

